# The Youngest Reported and Successfully Treated Patient with a Dermoid Cyst of the Parotid Gland: A Rare Pediatric Case

**DOI:** 10.1155/2017/4187030

**Published:** 2017-07-16

**Authors:** Marcel Fabian Glaas, Jörg Schipper, Nelofar Kajasi, Angelika Albrecht

**Affiliations:** ^1^Department of Otorhinolaryngology, Düsseldorf University Hospital (UKD), Duesseldorf, Germany; ^2^Department of Pathology, Düsseldorf University Hospital (UKD), Duesseldorf, Germany

## Abstract

Dermoid cysts (DCs) are rare benign, epithelial-lined lesions. Up to 7% of them are found in the head and neck region and 80% of those predominantly occur in the orbit, in the nose, and in the floor of the mouth. The average age of presentation is around the age of six. Dermoid cysts located in the parotid gland have only been published in 19 cases so far. Interestingly, the mean age of occurrence in the parotid gland was much higher (31 years). We report on a four-year-old girl being the youngest patient who had ever been diagnosed with this disease.

## 1. Introduction

A dermoid cyst (DC) is defined as a closed, epithelium-lined cavity in the body that contains differentiated tissue and structures like hair, fluid, teeth, or skin glands [1]. Dermoid cysts are a result of the inclusion of epithelial tissue during embryogenesis. Histologically, they are benign tumors that contain elements of the two germ layers ectoderm and mesoderm [2]. After the coccyx and the ovary, the head and neck region is the third most frequently affected area [1]. It accounts for 7% of all dermoid cysts. Of DCs in the head and neck region, up to 80% develop in the orbit, in the nose, and in the floor of the mouth. Malignant transformation is exceedingly rare and was mostly seen in the floor of the mouth. The literature reports on only 19 cases of dermoid cysts in the parotid gland. Most patients were adults with a mean age of 31 years at the time of diagnosis and only one case occurred in a nine-year-old child. Like other benign tumors in the parotid gland, DCs do not cause any specific symptoms and stay asymptomatic until they cause pressure-induced or cosmetic problems.

## 2. Case Report

A four-year-old girl was presented to our clinic with a 2-year history of a swelling of her left preauricular region. She did not complain of any prior disease, surgery, or trauma related to this region. The girl was otherwise healthy, with fever, lymphadenopathy, or weight loss being denied.

**Figure 1:**
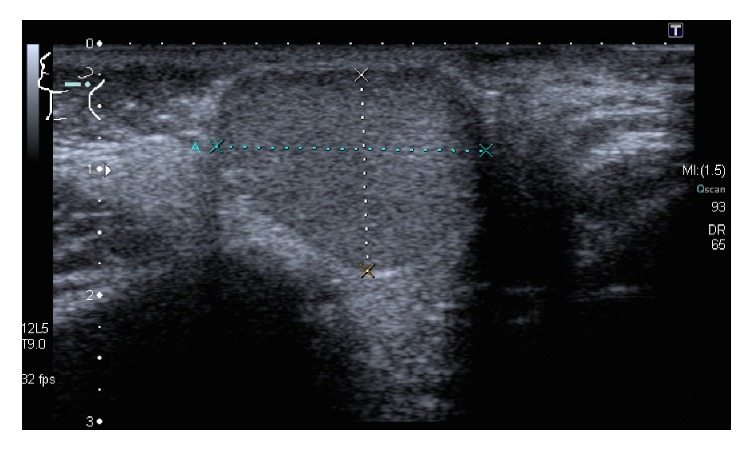
Ultrasonography of the left parotid gland showing a well-defined, homogenic, round, and hypodense mass with a diameter of 2.5 × 2 × 1.9 cm.

**Figure 2:**
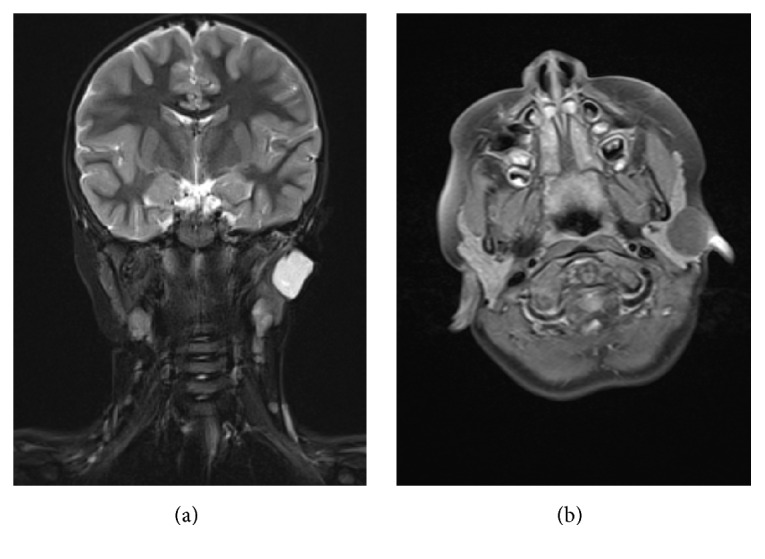
(a) Coronal MRI scan showing a signal enrichment in T2 weighting in the left parotid gland. (b) T1 weighted axial MRI scan showing a hypointensity of the same structure.

On physical examination, a painless, firm, and nonfluctuant mass was palpable in the area of her left parotid gland. Facial nerve function was normal on both sides. Ultrasonography showed a well-defined, homogenic, round, and hypodense mass with a measured diameter of 2.5 cm (Figure 1). The mass was embedded in the superficial lobe of the parotid gland and seemed to be capsulated. An MRI allowed a better preoperative evaluation referring to anatomy and dignity (Figures 2(a) and 2(b)). While the mass was not infiltrating into the surrounding tissue, it was, however, in close neighborhood to the external auditory canal. It showed a signal enrichment in T2 weighting (Figure 2(a)) and an intermediate signal in T1 (Figure 2(b)). It did not show any enhancement of the contrast medium. Pathological lymph nodes were also not detected.

**Figure 3:**
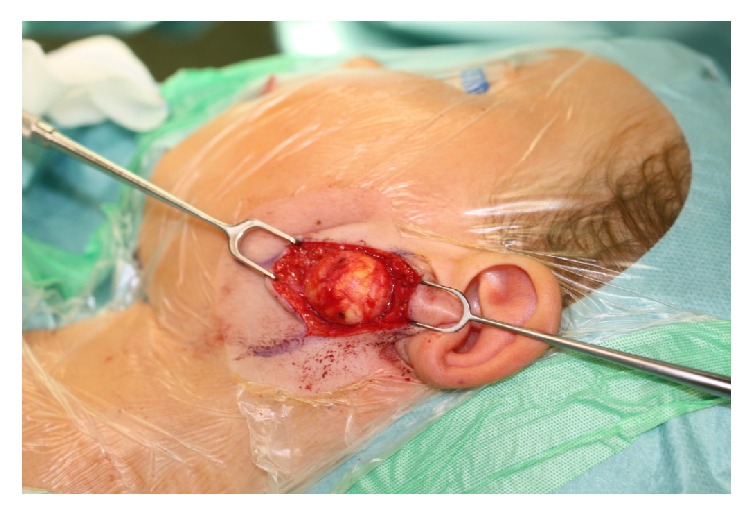
The intraoperative impression suggested a benign lesion with a tight capsule without abnormal vascularization.

Therefore, as the mass did not meet any criteria of malignancy in ultrasonography and MRI, we did not perform a fine-needle aspiration cytology (FNAC). A tumor resection was completed with a partial, superficial parotidectomy under continuous facial nerve monitoring. Intraoperatively, the tumor had a tight capsule without abnormal vascularization (Figure 3) and it was removed with parts of the adjacent parotid tissue.

**Figure 4:**
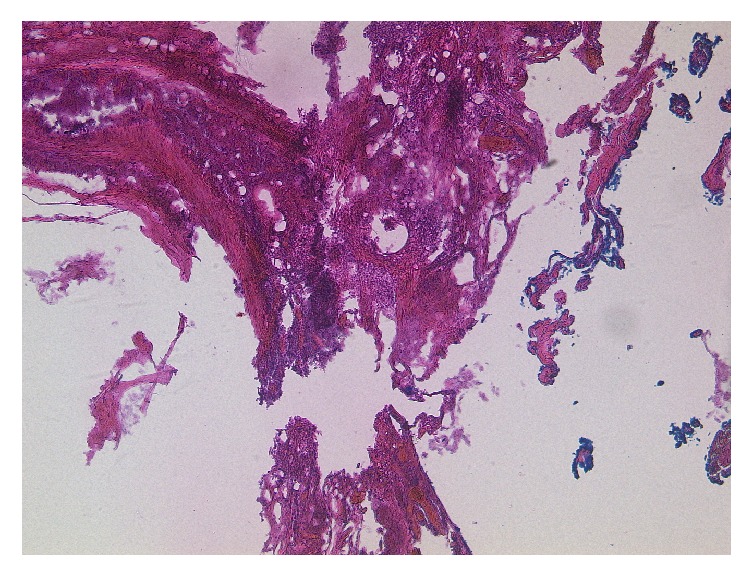
Dermoid cyst with stratified epithelium with associated skin appendages like keratin lamellae and sebaceous glands (haematoxylin and eosin staining).

Pathological examination showed a benign cyst with lining of squamous cell epithelium and associated skin appendages as it is typical for dermoid cysts. The lumen of the cyst contained several coreless squamous cells and keratin debris. The wall of the cyst contained fibrocartilaginous tissue with mild lymphocyte infiltration (Figure 4). An infiltration of lymphocytes, neutrophil granulocytes, and older adiponecrosis was found in parts of the underlying stroma.

In summary, the histology confirmed the diagnosis of a dermoid cyst. Postoperatively, the patient did show neither any facial nerve impairment nor any signs of recurrence in our follow-up.

## 3. Discussion

Only 7% of all dermoid cysts (DCs) are seen in the head and neck region and most of the children are about six years old at the time of diagnosis. Around 80% of DCs in the head and neck area are to be found in the orbit, nose, and floor of the mouth [3]. Few cases of malignant transformation have been reported, but only of DCs in the oral cavity [4]. While cystic lesions in the parotid gland make up 2–5% of all parotid gland tumors, dermoid cysts in the parotid gland are extremely rare. This article presents the 20th case worldwide and only the second one in a child [1, 5, 6]. Interestingly, the mean age of dermoid cysts of the parotid gland is higher (31 years) than in all other locations [1]. In a first systematic review of parotideal dermoid cysts, Yigit et al. recognized an increased occurrence in male patients with 76% (13/17), although the collective was small. As DCs rarely occur in the head and neck, a safe preoperative diagnosis is difficult.

Like other benign tumors of the parotid gland, DCs do not cause any specific symptoms and stay asymptomatic until they cause pressure-induced or cosmetic problems. Radiological imaging may guide the way. Ultrasound typically shows a well-circumscribed tumor with mixed or pseudosolid density (Figure 1). An MRI with i.v. contrast medium usually presents a cystic mass, which is typically hyperintense in T2-weighted images (Figure 2(a)) and hypointense in T1-weighted images (Figure 2(b)). A CT scan, which we did not perform, may also show a cystic lesion with a homogenous, hypodense content [7]. Although fine-needle aspiration cytology (FNAC) may provide diagnostic information of the cystic lesions, the preoperative importance and reliability are controversial [6, 8]. We decided not to perform a FNAC after considering the patient's age, the tumors appearance of being benign, and the necessity to remove the lesion in any case.

In addition, there are several possible differential diagnoses, including lipoma, lymphoepithelial cysts, fibroma or neurofibroma, blockage of parotid duct, or any kind of benign salivary gland tumor. As first branchial cleft anomalies are another possible differential diagnosis, it is recommended to look carefully for a sinus opening within the external ear canal or around the ear [9].

Although there have been several suggestions for a classification of dermoid cysts in the parotid gland, no agreement exists due to their rarity [3, 10]. While some authors simply categorize DCs in terms of congenital and acquired, New and Erich first classified them into three groups: (1) congenital DCs of the teratoma type; (2) acquired DCs; and (3) congenital inclusion DCs. Furthermore, they subdivided the third group into four subgroups: cysts around (a) eyes and orbits; (b) nose; (c) floor of mouth and submental and submaxillary region, and (d) suprasternal, suboccipital, thyroidal, lower lip, and palate region [3].

Choi et al. attribute DCs of the parotid gland to the third subgroup of the third category, while Yigit et al. hypothesize that these cysts might belong to the second category [1, 10]. They justify this classification with several arguments that our case disproves. We disagree that the higher frequency of DCs in adult age group supports the noncongenital theory as our patient was a four-year-old girl. Furthermore, there was—also considering the patient age—no history of a prior ear or parotideal surgery, which could have led to a traumatic tissue implantation.

Independent of its histopathological categorization, superficial parotidectomy is the standard procedure to remove the tumor. Total parotidectomy is only performed in rare cases of DCs that are located in deeper parts of the parotid gland [1]. Even though malignant transformation of DCs has been reported for those of the oral cavity, a complete surgical excision is state of the art [4]. Dermoid cysts have a tight capsule, which, as known of other parotid gland tumors, should not be harmed, as residual tissue may cause recurrence.

## 4. Conclusion

Dermoid cysts are benign tumors that rarely occur in the parotid gland. Although malignant transformation in the head and neck region has only been described in DCs of the floor of the mouth, they should be completely removed by microscopic surgery under continuous facial nerve monitoring. Preoperative imaging via ultrasonography and MRI or a preoperative fine-needle aspiration cytology can provide diagnostic clues. However the definitive diagnosis can only be confirmed by histopathological examination.
